# Hyperspectral Microscopy of Near-Infrared Fluorescence Enables 17-Chirality Carbon Nanotube Imaging

**DOI:** 10.1038/srep14167

**Published:** 2015-09-21

**Authors:** Daniel Roxbury, Prakrit V. Jena, Ryan M. Williams, Balázs Enyedi, Philipp Niethammer, Stéphane Marcet, Marc Verhaegen, Sébastien Blais-Ouellette, Daniel A. Heller

**Affiliations:** 1 Memorial Sloan-Kettering Cancer Center, New York, NY, USA; 2 Weill Cornell Medical College, New York, NY, USA; 3 Photon Etc., Montreal, Canada

## Abstract

The intrinsic near-infrared photoluminescence (fluorescence) of single-walled carbon nanotubes exhibits unique photostability, narrow bandwidth, penetration through biological media, environmental sensitivity, and both chromatic variety and range. Biomedical applications exploiting this large family of fluorophores will require the spectral and spatial resolution of individual (n,m) nanotube species’ fluorescence and its modulation within live cells and tissues, which is not possible with current microscopy methods. We present a wide-field hyperspectral approach to spatially delineate and spectroscopically measure single nanotube fluorescence in living systems. This approach resolved up to 17 distinct (n,m) species (chiralities) with single nanotube spatial resolution in live mammalian cells, murine tissues *ex vivo*, and zebrafish endothelium *in vivo*. We anticipate that this approach will facilitate multiplexed nanotube imaging in biomedical applications while enabling deep-tissue optical penetration, and single-molecule resolution *in vivo*.

In biological imaging applications, multi-color imaging is often limited by large spectral bandwidths, photobleaching interactions, and interference from the intrinsic optical properties of tissues^1^,^2^. Semiconducting single walled carbon nanotubes (SWCNTs) exhibit intrinsically photostable^3^ excitonic fluorescence^4^ in the near-infrared (nIR) region of the spectrum (800–1700 nm)—a region which facilitates biological imaging due to attenuated tissue absorbance, scattering, and autofluorescence^1^,^5^. Carbon nanotube fluorescence responds to its environment via changes in intensity, wavelength, or spectral bandwidth^6^. Nanotube-based optical sensors have been developed to detect various analytes including small molecules^7^, oxidative radicals^8^, and macromolecules^9^. Nanotube emission bands corresponding to each unique (n,m) species (chirality) are narrow (~20 nm)^10^ compared to organic fluorophores^2^, allowing a larger number of spectrally-separated emitters to be imaged simultaneously. Recently, nanotubes have been fluorescently imaged in brain blood vessels of live mice^11^, used for *in vivo* long term sensing^12^, and deployed as fluorescent markers for surgical tumor resection at depths up to 18 mm^13^. However, these imaging-based applications treated the entire family of emissive nanotubes as a single fluorophore, even though as many as 33 spectrally different fluorescent species exist^10^.

Spectral imaging is a powerful tool for detection, validation, separation, and quantification in applications ranging from mineral assessment of geological satellite images^14^ to semiconductor material characterization^15^. In contrast to multi-spectral imaging of discrete wavelength bands, hyperspectral imaging produces a continuous emission spectrum at every spatial pixel^16^. A recent approach to spectral imaging, termed global hyperspectral imaging, uses volume Bragg gratings (VBG)^17^,^18^ to acquire spectrally-defined images from the scanned wavelength space. This method has been applied for the mapping of solar cell saturation currents and in astronomical imaging^19^,^20^.

Herein, we developed a wide-field near-infrared hyperspectral microscopy approach to spatially observe the fluorescence and spectral heterogeneity from single nanotubes in complex environments, including live cells and tissues. Exploiting the narrow spectral bandwidth (full width at half maximum, FWHM) of nanotubes, we resolved 17 distinct chiralities of individual nanotubes on a surface using a single excitation laser. In live cells, 12 distinct fluorescent nanotube species were simultaneously detected in a 500 nm imaging window. We used this approach to spectrally image and identify the chiralities of individual nanotubes in mouse tissue *ex vivo* and within zebrafish embryos *in vivo*. This approach is the first to spatially identify multiple nanotube photoluminescence emission bands in living systems and the first measurement of single nanotube spectra in biological specimens. The work portends the use of the family of photoluminescent carbon nanotube probes, as well as other nIR fluorescent materials such as quantum dots^21^,^22^, for multiplexed biomedical imaging.

We constructed a near-infrared hyperspectral microscope by incorporating a volume Bragg grating between the emission port of a standard inverted fluorescence microscope and the nIR camera (Fig. 1a) to obtain the spectral properties of the spatially-resolved emitted light. By specifying the angle (θ) of a turret-mounted grating with respect to the collimated emission beam from the sample (λ_All_), a ray of center wavelength λ_B_ = 2n Λsin(θ) is reflected by the grating into the detector, where n is the refractive index and Λ is the period of the grating (Fig. 1b). The emission was passed twice through the volume Bragg grating which resulted in a reduced bandwidth (Fig. 1c). A continuous stack (hyperspectral cube) of 152 images (256 × 320 pixels) with a 3.7 nm bandwidth was obtained between 844 to 1452 nm, collected in 4 nm steps, and rectified to create a stack of 126 images between 900 to 1400 nm (described further in the Supplementary Information). The integration time for each of the 152 images generally ranged from 0.05–4 s, dependent upon signal, which resulted in hyperspectral cube acquisition times between 20 s and 10 min (Table S1).

We used hyperspectral imaging to resolve the chiralities of single nanotubes adsorbed to a surface. Single-walled carbon nanotubes (Rice HiPco preparation) were suspended with sodium deoxycholate (SDC). Excess surfactant was removed by centrifugal filtration. The SDC-HiPco nanotubes were adsorbed onto a poly-D-lysine coated glass surface and dried with ultra-pure N_2_ for imaging in air (Supplementary Methods). A nIR broadband (900–1500 nm) image (Fig. 2a) and a hyperspectral cube of nanotube photoluminescence were acquired under 730 nm excitation at 230 mW power at the sample (Movie S1). Excitation from the multi-mode laser fiber delivery was randomly polarized, and the total power measured on the sample did not vary with the polarization angle (Table S2). Individual nanotubes, excited by the laser, appeared as bright fluorescent objects. Rotation of a quarter-wave plate inserted into the excitation path did not modulate the emission intensity from individual nanotubes (Fig. S1). One would expect modulation in the case of a polarized excitation beam due to the angle dependence of the wave plate. In addition, the nanotube emission was photostable under these experimental conditions (Fig. S2). The emitted spectrum of each nanotube, with an average signal-to-noise ratio of greater than 900 (Fig. S3), was fit with a Voigt function to determine the intensity, center wavelength, and spectral bandwidth (FWHM). We changed the exposure time from 0.05 to 1 s and found that, while peak intensity scaled monotonically with exposure time, the fitted center wavelengths from a single nanotube were within 1 nm (st. err = 0.35 nm), indicating that the nanotube emission peak was independent of the emitted intensity (Fig. S4). The center wavelengths corresponding to 892 imaged nanotubes were each assigned to one of 17 chiralities using a one-dimensional k-means clustering algorithm^23^ (Fig. 2b, S5). The chirality assignment facilitated the composition of a false-color photoluminescence image of a microscope field full of nanotubes (Fig. 2c) and the spectral discrimination of 17 nanotube species within a 500 nm window (Fig. 2d). By counting nanotubes of each species, we produced the chirality distribution of the sample (Fig. 2e), which closely approximated the solution ensemble spectrum (Fig. 2f, S6). Upon analyzing the mean peak intensity, center wavelength, and bandwidth for each chirality, we found a strong correlation between peak energy and FWHM (Figs. 2g, 0.94 Pearson’s correlation, p = 4·10–6). Individual nanotubes with longer emission wavelengths exhibited narrower FWHMs, consistent with the correlation observed when nanotubes were grown directly on a surface^24^. Nanotubes that appeared longer exhibited brighter emission (Fig. S7), corroborating previous studies^25^.

The hyperspectral imaging approach allowed the resolution of single carbon nanotube spectra in biological environments, permitting the identification of chiralities. We incubated human cervical cancer cells (HeLa CCL-2) with 1 mg/L SDC-encapsulated HiPco nanotubes for 30 minutes at 37 °C, before washing away free nanotubes prior to imaging. Single nanotubes appeared as distinct, punctate fluorescent objects (Fig. 3a,b) with a median distance of 7 μm between each ROI and its nearest neighbor, suggesting that each ROI consisted of only one endosome (Fig. S8). Spectra of the cells, acquired using a conventional spectrometer and nIR detector, showed the presence of 5 broad emission peaks which were difficult to resolve into the emission bands of individual chiralities (Fig. 3c). We confirmed that our nanotube complexes were internalized by energy-dependent endocytic mechanisms and remained in the endolysosomal pathway via incubation at 4 °C (Fig. S9) and co-localization of nanotubes with the Lysotracker endosomal stain (Fig. S10), similar to SWCNTs encapsulated in other anionic coatings^26^,^27^. In live HeLa cells, we detected 12 different nanotube (n,m) species to produce a chirality-mapped false-color image (Fig. 3d,f, Table S3, Fig. S11, Movie S2). The imaging and assignment of 12 different nanotube emitters using this far-field epifluorescence microscopy technique did not require spectral deconvolution.

Nanotube photoluminescence in the near-infrared emission window (900–1400 nm) afforded the multiplexed detection of 8 nanotube chiralities in murine tissues *ex vivo*. Sodium cholate-suspended nanotubes were injected subcutaneously into a hairless SKH1 mouse. Dermal tissue was harvested after 30 minutes, formalin-fixed, and paraffin-embedded for imaging (Supplementary Methods). An overlay of transmitted light and nIR fluorescence images of a vertical cross-section shows a diffuse pattern consistent with nanotube uptake and distribution in adipocytes (Fig. 4a). In the DAPI-stained transmitted-light image of a horizontal tissue cross-section (Fig. 4b), we used hyperspectral imaging to detect and assign chiralities to individual nanotubes (Fig. 4c). The different nanotube chiralities were easily distinguished (Fig. 4d), and the imaging benefitted from the decreased tissue auto fluorescence in this spectral region^28^, resulting in an average signal-to-noise of over 40 (Fig. S12).

The *in vivo* imaging of single fluorescent nanotubes was accomplished within an anesthetized zebrafish. DNA-encapsulated nanotube complexes were introduced to 3 day old zebrafish embryos via cardinal vein injection. The zebrafish were imaged within 30 minutes of injection, after immobilization within agarose. Hyperspectral cubes of a live zebrafish tail section were acquired (Fig. 4e); imaging with 730 nm excitation (230 mW power at the sample) did not cause noticeable morphological change to the embryos. The spectrally-defined images show nanotubes within cells of the zebrafish vessel wall, confirmed by co-injection of FITC-labeled dextran (Fig. 4f). Single nanotubes were imaged *in vivo* (Fig. 4g), and their chiralities were assigned from the corresponding emission spectra (Fig. 4h, mean SNR > 40). A movie of the nIR emission within the live animal suggests that the nanotubes were taken up by endothelial cells (Movie S3).

In this work, a hyperspectral imaging approach was developed to observe chirality-resolved, single nanotube photoluminescence in living cells and tissues. Taking advantage of the narrow-band emission of nanotubes, simultaneous multicolor imaging was used to resolve 17 nanotube chiralities, including 12 distinct fluorescent species within live cells. We imaged and identified single nanotubes by chirality both in mouse tissue *ex vivo* and *in vivo*, within live zebrafish embryos. This approach enabled biological imaging with a greatly expanded set of near-infrared fluorophores whose properties allow greater multiplexing, higher depth penetration into tissues, unique photostability, and single-fluorophore resolution at far field.

## Additional Information

**How to cite this article**: Roxbury, D. *et al.* Hyperspectral Microscopy of Near-Infrared Fluorescence Enables 17-Chirality Carbon Nanotube Imaging. *Sci. Rep.* **5**, 14167; doi: 10.1038/srep14167 (2015).

## Supplementary Material

Supplementary Information

Supplementary Movie S1

Supplementary Movie S2

Supplementary Movie S3

## Figures and Tables

**Figure 1:**
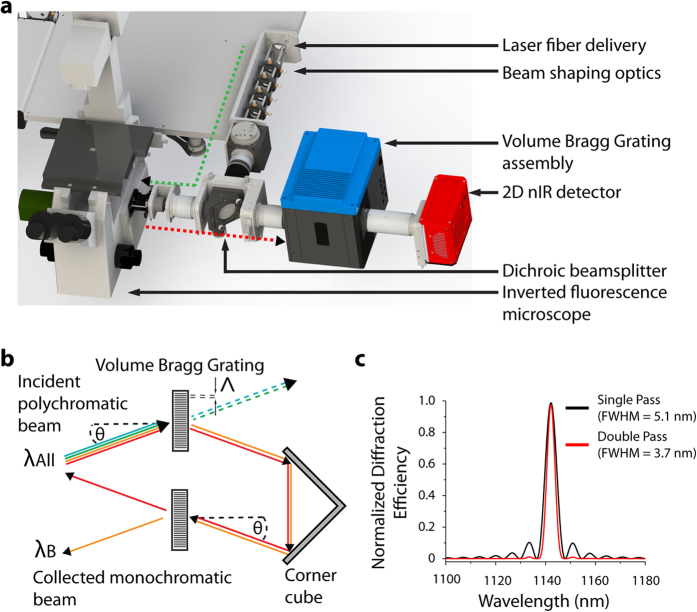
Near-infrared fluorescence hyperspectral microscope. (**a**) A reconstruction of the hyperspectral imaging microscope indicating the injection of the excitation laser (green) into the inverted microscope assembly and the nIR emission from the sample (red), collected by the 2D nIR InGaAs detector via the volume Bragg grating (VBG). (**b**) Schematic of the VBG: a specific wavelength component of the incident polychromatic light λ_All_ is diffracted by the grating as a function of incident angle θ, refractive index n, and grating period Λ, while the remaining wavelengths are transmitted through the grating. After a second passage through the VBG, a monochromatic beam λ_B_ exits. (**c**) Normalized diffraction efficiency after one (red) and two (black) passes through the VBG for λ_B_ equal to 1142 nm.

**Figure 2:**
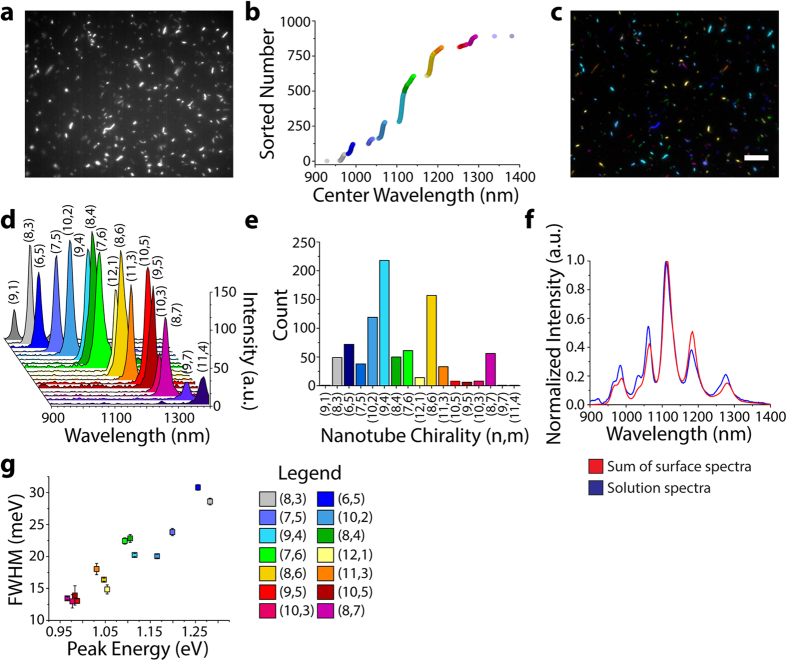
Hyperspectral microscopy of carbon nanotubes on a surface. (**a**) A nIR broadband (900–1500 nm) fluorescence image of SDC-suspended HiPco carbon nanotubes adsorbed to a glass surface. (**b**) The center wavelengths from fitted emission spectra obtained from hyperspectral cubes, sorted in ascending order. (**c**) A false-color image of the same region as shown above, colored by nanotube chirality. Scale bar, 10 μm. (**d**) A representative spectrum of a single nanotube of each of the 17 species detected in a 500 nm emission window. (**e**) The total population of each nanotube species summed from hyperspectral cubes of 10 different 90 μm by 70 μm regions. (**f**) The population distribution derived from the spectral sum of the 17 Gaussian distributions (red) closely approximated the bulk solution spectrum obtained with the same excitation wavelength (blue). (**g**) Photoluminescence spectral bandwidth of nanotube chiralities, plotted by emission energy; error bars denote standard error of the mean.

**Figure 3:**
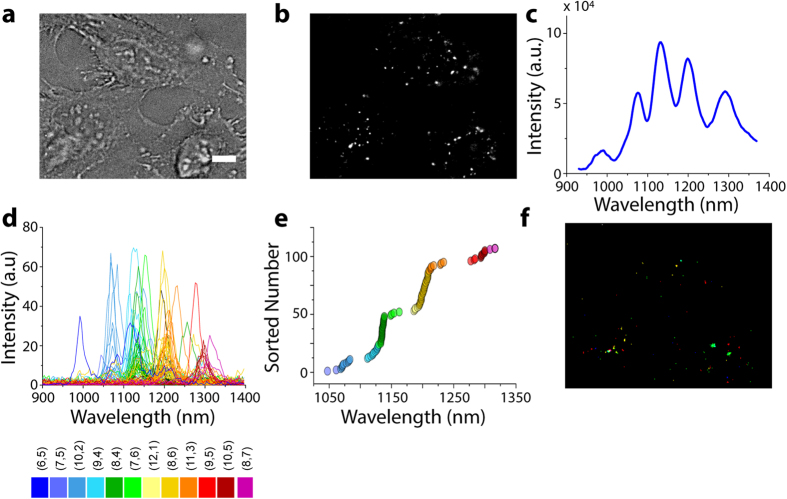
Hyperspectral microscopy of individual carbon nanotubes in live mammalian cells. (**a**) Transmitted light image of live HeLa cells incubated with SDC-dispersed nanotubes for 30 minutes. Scale bar, 10 μm. (**b**) Broadband nIR image (900–1600 nm) of the same region. (**c**) Near-infrared spectrum of carbon nanotubes from within HeLa cells as acquired from a conventional spectrometer/nIR detector. (**d**) Spectra from within the live HeLa cells, acquired from 10 cubes of hyperspectral data. (**e**) Fitted emission peak wavelength values sorted in ascending order by emission wavelength. (**f**) False-colored image of the same region as shown in (**b**), colored by nanotube chirality.

**Figure 4:**
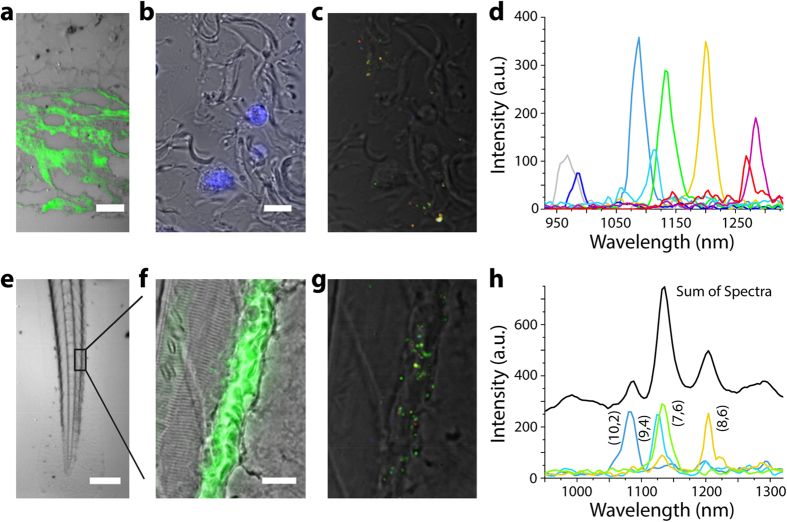
Hyperspectral microscopy of carbon nanotubes *ex vivo* and *in vivo*. (**a**) A vertical cross section of formalin-fixed and paraffin-embedded dermal tissue harvested from a mouse after subcutaneous injection of carbon nanotubes. Scale bar, 50 μm. (**b**) Horizontal cross section of tissue stained with DAPI (blue). Scale bar, 10 μm. (**c**) Spectrally-resolved nIR emission merged with a transmitted light image in horizontal cross section. (**d**) Spectra of 8 individual species of nanotubes present in the imaging field. (**e**) Transmitted light image of an anesthetized zebrafish injected with nanotubes via the common cardinal vein. Scale bar, 200 μm. (**f**) Magnified transmitted light image of zebrafish tail fin and FITC-500 kDa-dextran fluorescence (green), delineating blood vessels. Scale bar, 20 μm. (**g**) Spectrally-resolved nIR emission overlaid with a transmitted light image of the same region. (**h**) Representative spectra of 4 individual nanotube species found in the zebrafish caudal vein. The majority of bright regions contained multiple co-localized nanotubes (black curve). Since the (7,6) nanotube species was the dominant peak in these aggregates, a high prevalence of green is found in the color-coded nIR emission image.
