# Supplementary material for: Hyperspectral Microscopy of Near-Infrared Fluorescence Enables 17-Chirality Carbon Nanotube Imaging
Source: Sci Rep. 2015 Sep 21;5:14167. doi: 10.1038/srep14167 (PMC4585673; doi:10.1038/srep14167)
Supplement: Supplementary Information [file srep14167-s1.pdf]

Supplementary Materials for

Hyperspectral Microscopy of Near-Infrared Fluorescence Enables 17-Chirality Carbon Nanotube Imaging

Daniel Roxbury[1†], Prakrit V. Jena[1†], Ryan M. Williams[1], Balázs Enyedi[1], Philipp Niethammer[1,2], Stéphane Marcet[3], Marc Verhaegen[3], Sébastien Blais-Ouellette[3], and Daniel A. Heller[1,2]*

Affiliations:

[1]Memorial Sloan-Kettering Cancer Center, New York, NY, USA.

[2]Weill Cornell Medical College, New York, NY, USA.

[3]Photon Etc., Montreal, Canada.

*Correspondence to: hellerd@mskcc.org

†Contributed equally to this work

Materials and Methods

Hyperspectral Microscope System Excitation: A continuous wave (CW) 730 nm diode laser with an output power of 1 W was injected into a multimode fiber to produce the excitation source for photoluminescence experiments. To ensure a homogenous illumination over the entire microscope field of view, the excitation beam passed through a custom beam shaping module to produce a top hat intensity profile within 20% variation on the surface of the sample under test. This top hat profile effectively overfills the imaging field of view with the top flat part of the hat. A long pass dichroic mirror with a cut-on wavelength of 880 nm was aligned to reflect the laser into an Olympus IX-71 inverted microscope (with internal optics modified for near infrared transmission) equipped with a 100X (UAPON100XOTIRF, NA=1.49) oil, 50x (LCPlan N, NA=0.65, IR), or 20x (LCPlan N, NA=0.45, IR) objective (Olympus, USA).

Hyperspectral Filter Construction: The hyperspectral filter was situated in the system between the long-pass dichroic mirror and the camera. To decrease the amount of photons produced by elastic laser scattering at the surface of the sample, a long-pass filter with cut-on wavelength of 815 nm was placed at the filter optical input. Fig. BTF illustrates the conceptual view of the hyperspectral filter. For simplicity, only a single beam produced by a point source in the field of view of the microscope is shown. To ensure that the beam entirely passed through the volume Bragg grating (VBG) for filtering, the pupil of the optical system was imaged between two VBG passes by the first tube lens. Non-resonant light passes through a VBG un-diffracted. Only the wavelength that complies with the Bragg condition will resonate with the grating and be diffracted in the direction of the corner cube. The corner cube was situated to reflect the filtered beam onto the VBG for a second pass in order to cancel chromatic dispersion induced by the first pass and to narrow filtered bandwidth to 3.7 nm (Fig. 1c, main text). The second tube lens formed a spectrally-filtered image on the IR camera sensor. After two passes through the VBG, the combined efficiency was wavelength dependent, and measured between 40 and 60%.

To acquire spectrally filtered images at different wavelengths, the corner cube and the VBG were positioned on rotation stages to continuously tune the diffracted wavelength. An automatic wavelength calibration process was designed to guarantee the relation between the angle of the rotation stage and the absolute wavelength of the grating. Using a xenon spectral lamp (Newport 6033), absolute wavelength accuracy of 0.5 nm was obtained.

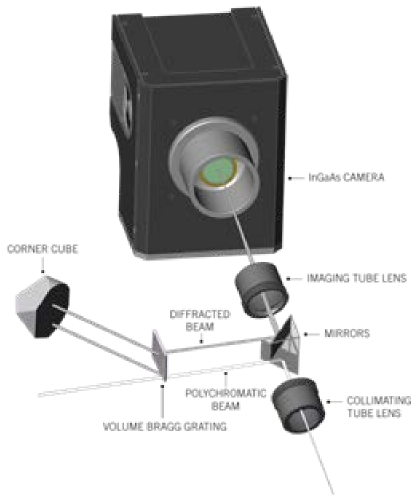

Figure BTF. Conceptual view of a transmission imaging Bragg tunable filter.

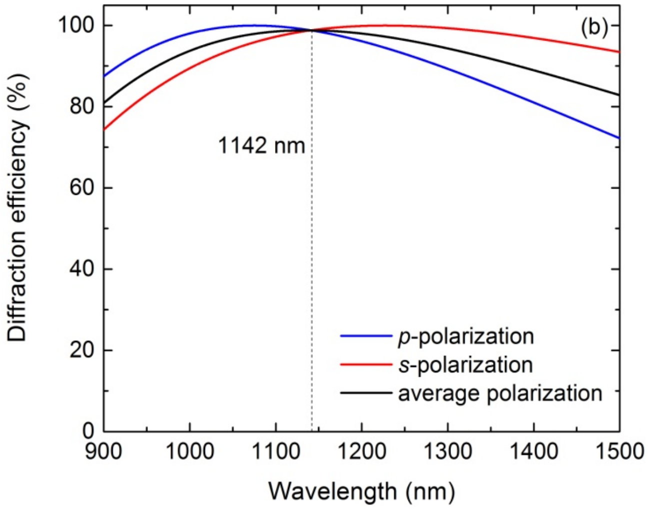

Figure DE. Spectral response of the volume Bragg grating over the whole spectral range for *p*-, *s*-, and average polarization (blue, red, and black lines, respectively). The maximum spectral response for average polarization state falls at 1142 nm.

Hyperspectral Microscope System Camera: A deep-cooled short-wave infrared (SWIR) camera was designed for the hyperspectral system. A 2D nIR detector (InGaAs sensor array) operational between 900 nm and 1700 nm and with a quantum efficiency greater than 70% over the entire range was used. The array, consisting of 320x256 pixels with 30 µm pitch, was coupled with a four-stage TE cooler to keep the sensor operating temperature below 190 K. The full-well capacity of the camera was 168000 electrons in high gain and 3.5 million electrons in

low gain with 65dB S/N ratio and 346 frames per second capability (full frame). The dynamic range was 14 bits and the readout noise was 57 electrons at 346 fps in high gain. Images in the visible (400 – 700 nm) range were acquired using a QIClick digital CCD camera (Qimaging, Surrey, BC, Canada) attached to a separate port of the microscope.

Hyperspectral Cube Rectification: Each point source produced a collimated beam having a different incident angle on the VBG, as an image is a sum of point sources issued from different positions of the object seen by the microscope objective. Therefore, the angular selectivity of the grating resulted in a gradient in wavelength across the field of view in the dimension parallel to the dispersion axis (Fig. GRADIENT). The filtered image produced on the InGaAs camera was composed of a series of vertical lines each with a specific wavelength. To obtain a monochromatic image, several frames at contiguous wavelengths must be scanned through in order to retrieve the wavelength of interest for each image. The reconstruction was performed using cubic interpolation on every pixel for each monochromatic image according to the wavelength calibration parameters.

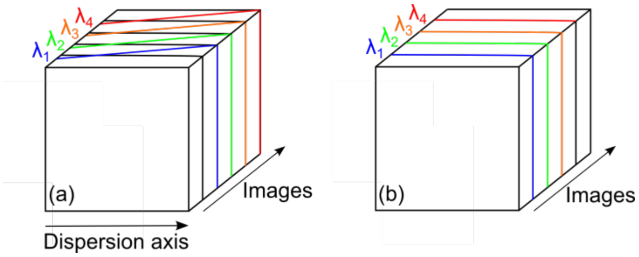

Fig. GRADIENT. (*A*) Images with a gradient in wavelength in the dimension parallel to the dispersion axis and (*B*) reconstructed monochromatic images after rectification.

Nanotube Sample Preparation:
Standard chemical reagents were purchased from Sigma-Aldrich (St.Louis, MO, US) and Fisher Scientific (Pittsburgh, US). Single-walled carbon nanotubes used throughout the study were commercially purchased and produced by the HiPco process (Unidym, Sunnyvale, CA or Rice University). Aqueous dispersions were created by the probe-tip ultrasonication (Sonics & Materials, Inc.) of 2 mg of the specified oligonucleotide (IDT DNA, Coralville, IA) or 20 mg sodium deoxycholate, SDC, with 1 mg of raw SWCNTs in 1 mL of deionized water for 30 minutes or 10 seconds at 40% of the maximum amplitude (~13 Watts). Following sonication, the dispersions were ultracentrifuged (Sorvall Discovery 90SE) for 30 minutes at 280,000 x *g*. The top ¾ of the resultant supernatant was collected and its concentration was determined with a UV/Vis/nIR spectrophotometer (Jasco, Tokyo, Japan) using the extinction coefficient A_{910} = 0.02554 $L{\cdot}mg^{-1}{\cdot}cm^{-1}$. To remove free DNA or SDC, 100 kDa Amicon centrifuge filters (Millipore) were used to concentrate the SDC-HiPco nanotube complexes, which were re-suspended in deionized water.

Cell Lines and Cell Culture Procedures: HeLa CCL-2 cells (ATCC, Manassas, VA) were grown under standard incubation conditions at 37˚C and 5% CO_2 in sterile-filtered DMEM with 10% heat-inactivated FBS, 2.5% HEPES, 1% Glutamine, and 1% Penicillin/Streptomycin (all Gibco). Cells were plated onto T-75 flasks at 20% confluence and passaged every 3 days.

For imaging experiments, cells were plated onto glass-bottom petri dishes (MatTek). Unpurified SWCNT were incubated at 1 mg/L and separated SWCNT were incubated at 0.25 mg/L in media with cells for 30 minutes at 37 °C. Depending on the experiment, cells were imaged immediately, or trypsinized (Gibco) and re-plated onto a fresh glass-bottom petri dish. If required, cells were fixed with a solution of 4% paraformaldehyde in PBS for 15 minutes, washed, and placed in fresh PBS for imaging.

Ex Vivo Mouse Imaging Protocol: Female SKH1 hairless, immune competent mice (Charles River, Wilmington, MA) were used in accordance with MSKCC Institutional Animal Care and Use Committee guidelines[1]. An approximately 2 month old SKH1 mouse was injected subcutaneously with 100 µL of 1 – 100 mg/L SDC-nanotubes in each flank. The mouse was sacrificed 30 minutes following injection. Dermal and subdermal layers in 5 mm squares surrounding the injection site were grossly dissected and fixed in 4% paraformaldehyde overnight. Un-injected tissues were obtained and identically processed for control images. The tissues were paraffin-embedded and sliced into 5 µm longitudinal and transverse sections. The sections were placed on a glass microscope slide with a cover slip for imaging.

In Vivo Zebrafish Imaging Protocol: Adult casper zebrafish[2] were maintained according to institutional animal guidelines as described[3]. The larvae (2.5-3 days post fertilization) were anesthetized with 0.2 mg/ml tricaine (Sigma). A 2.3 nL solution containing 1 mg/L ss$(AT)_{15}$-BSA-Hipco nanotubes (i.e. BSA added in excess with DNA-nanotubes, vortexed and subjected to centrifugal filtration with a 100 kDa MWCO (Amicon)), and 0.1 mg/ml 500 kDa FITC-dextran (Life Technologies) was injected into the common cardinal vein. The larvae were then placed on glass bottom dishes (MatTek Corporation) and embedded in 1% low melting agarose (Lonza) for imaging.

Experimental Data Acquisition: For surface-based measurements, SDC-nanotubes at 1 mg/L, were incubated on a poly-d-lysine coated glass-bottom 35 mm petri dish for 10 seconds and immediately withdrawn by pipette. While focusing on the surface, the individual SWCNTs were excited with a 730 nm laser, ~230 mW power at the sample. Hyperspectral cubes were acquired from 900 to 1400 nm using an exposure time of 2-4 s per image, depending on signal. Hyperspectral cubes from blank surfaces were also acquired for background subtraction.

Analysis Software: Hyperspectral data and wide field image acquisition, as well as cube rectification, were performed using the PhySpec software (Photon etc., Montreal, Canada). Data from hyperspectral cubes were analyzed by an automated region of interest (ROI)-picking algorithm. The mean intensity of a region devoid of ROIs was defined as the background signal. Subtracting the background value from each ROI resulted in an approximately zero baseline for the spectrum from each nanotube. Data analysis, curve fitting, and simulation programs were written in Matlab 2011 (The MathWorks, MA, US). Statistical analysis and graphs were generated using OriginPro 8.6 (OriginLab, Northampton, MA).

Photoluminescence Excitation/Emission Contour Plots: Photoluminescence (PL) plots were acquired using a home-built apparatus consisting of a tunable white light laser source, inverted microscope, and InGaAs nIR detector. The SuperK EXTREME supercontinuum white light laser source (NKT Photonics) was used with a VARIA variable bandpass filter accessory capable of tuning the output 500 – 825 nm with a bandwidth of 20 nm. A longpass dichroic mirror (900

nm) was used to filter the excitation beam. The light path was shaped and fed into the back of an inverted IX-71 microscope (Olympus) where it passed through a 20x nIR objective (Olympus) and illuminated a 200 µL nanotube sample in a 96-well plate (Greiner). Emission from the nanotube sample was collected through the 20x objective and passed through a dichroic mirror (875 nm, Semrock). The light was f/# matched to the spectrometer using several lenses and injected into an Isoplane spectrograph (Princeton Instruments) with a slit width of 410 µm which dispersed the emission using a 86 g/mm grating with 950 nm blaze wavelength. The light was collected by a PIoNIR InGaAs 640 x 512 pixel array (Princeton Instruments).

Excitation, emission, and wavelength corrections and calibrations were performed as follows. The power at each excitation wavelength was measured at the objective with a PM100D power meter (Thorlabs) from which a power spectrum was constructed and used to correct the emission intensities for non-uniform excitation. A HL-3-CAL-EXT halogen calibration light source (Ocean Optics) was used to correct for wavelength-dependent features in the emission intensity arising from the spectrometer, detector, and other optics. A Hg/Ne pencil style calibration lamp (Newport) was used to calibrate spectrometer wavelength.

Acquisition was conducted in automated fashion controlled by Labview code which iteratively increased the excitation laser source from 500 – 824 nm in steps of 3 nm, acquired data with an exposure time of 0.3 seconds for a nanotube concentration of 1 mg/L SDC-HiPco, and saved the data in ASCII format. The spectral range was 930 – 1369 nm with a resolution of ~0.7 nm. Background subtraction was conducted using a well filled with DI H_2O. Data acquisition was on the order of 1-2 minutes per sample, or ~2 hours per 96-well plate. Following acquisition, the data was processed with Matlab code which applied the aforementioned spectral corrections, created the contours with a Gaussian smoothing function, and constructed figures to be used for manual assignment of nanotube chiralities from the two-dimensional peaks.

Supplementary Figures

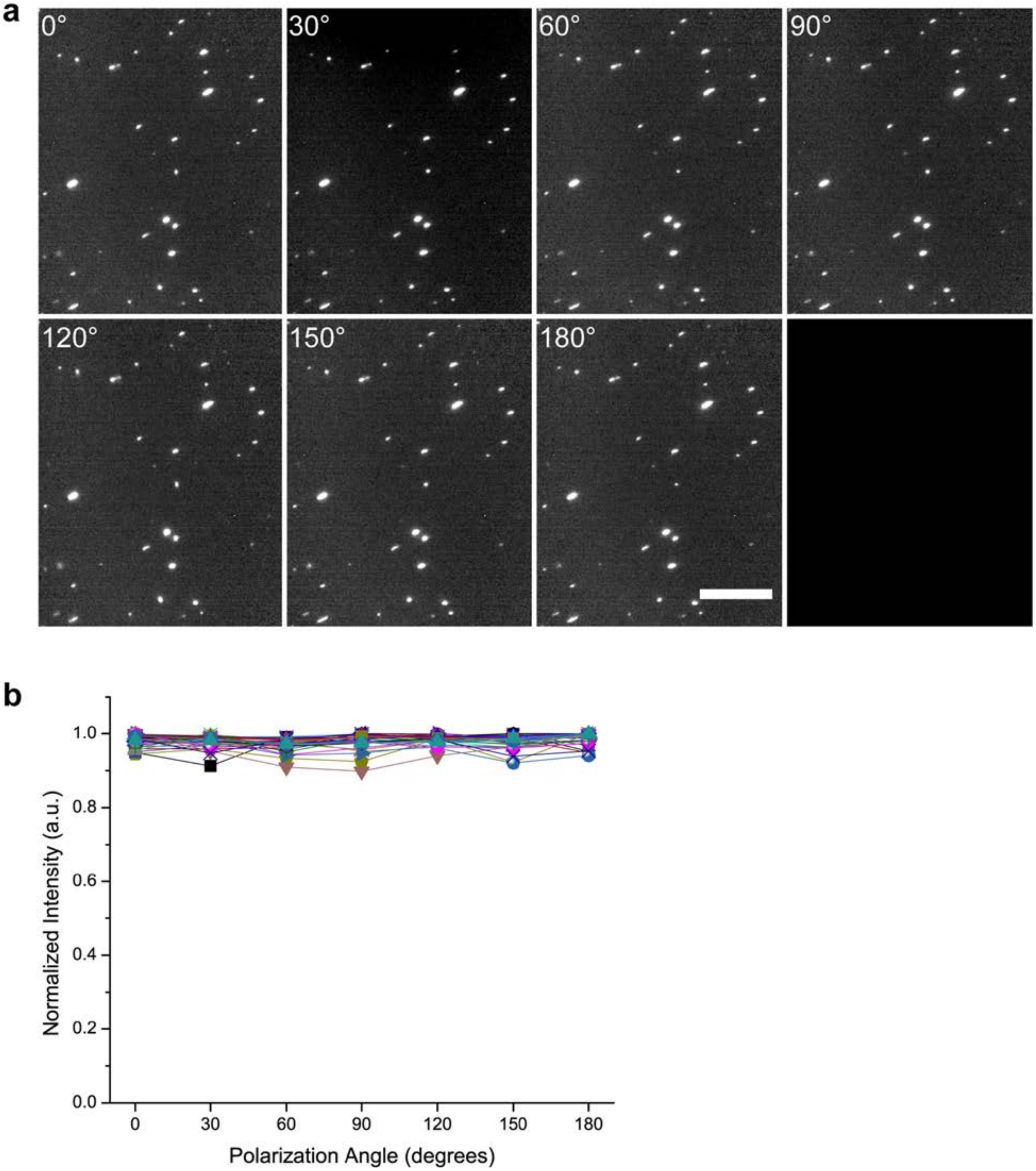

Figure S1. **Measurement of excitation source polarization. a**, Images of SDC-HiPco nanotubes adsorbed to a glass surface, set to the same brightness and contrast settings, acquired as a function of quarter-wave plate (QWP) angle rotated with respect to the laser axis. Scale bar is 20 µm. **b**, The averaged intensity of 35 individual nanotubes as a function of the angle between the QWP and the laser axis.

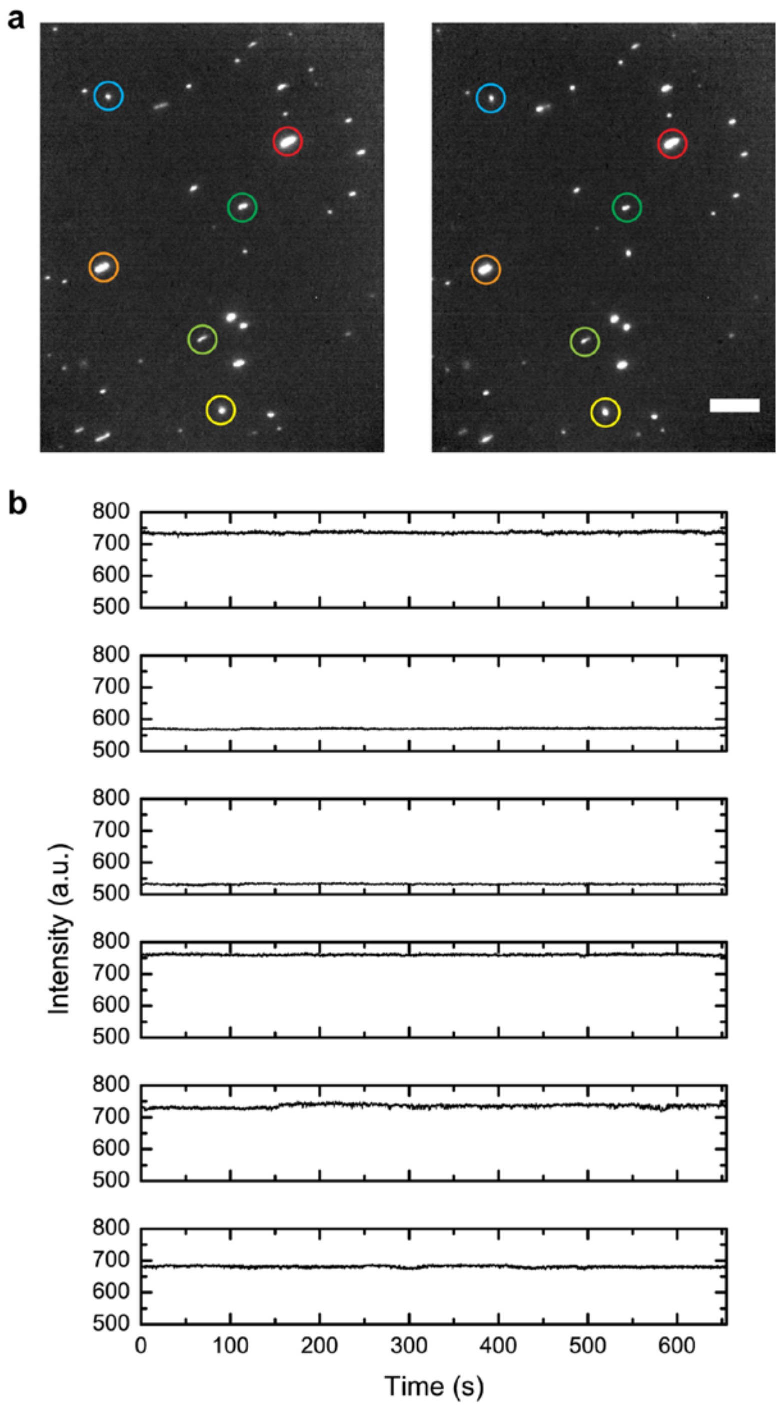

Figure S2. **Photostability of nanotube emission under continuous excitation. a**, Images of surface-adsorbed SDC-HiPco nanotubes, before and after acquiring a 12:55 minute movie—longer than the time required to acquire a 4s, 126 frame hyperspectral cube—using 730 nm laser excitation with 230 mW of power at the sample. Images are displayed with identical brightness and contrast settings. **b**, The intensity time traces of 6 individual nanotubes, showing complete photostability.

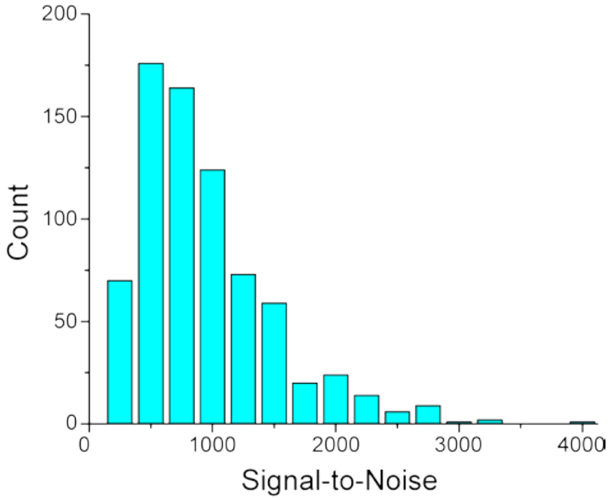

Figure S3. Spectral signal-to-noise ratio (SNR) was calculated for the sample as: SNR= [(Peak Height-Background) / St.Dev. of the background)] where background is the mean signal from the emission spectra ± 20 nm from the emission peak.

Sodium deoxycholate (SDC)-suspended HiPco nanotubes, adsorbed onto a glass surface, were imaged in air. After fitting each nanotube spectrum with a Voigt function, fits with an R^2 value of less than 0.9 were rejected from all following analyses. The emission peak value was divided by the standard deviation of the baseline to obtain the SNR for the particular nanotube. A distribution of the SNR for the sample was created to extract statistical parameters. The mean of the distribution was determined to be 941.17 ± 20.25 (standard error, n=892).

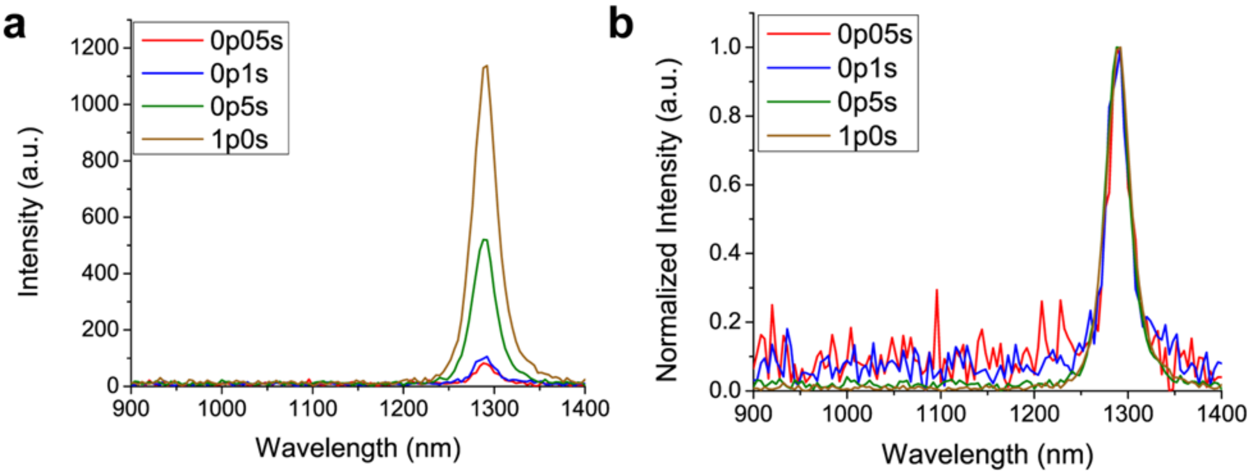

Figure S4. **Nanotube spectra as a function of exposure time. a**, Spectra of SDC-HiPco nanotubes, adsorbed to a glass surface, acquired at four different exposure times. **b**, Normalized spectra indicate that emission peak wavelength does not vary with exposure time.

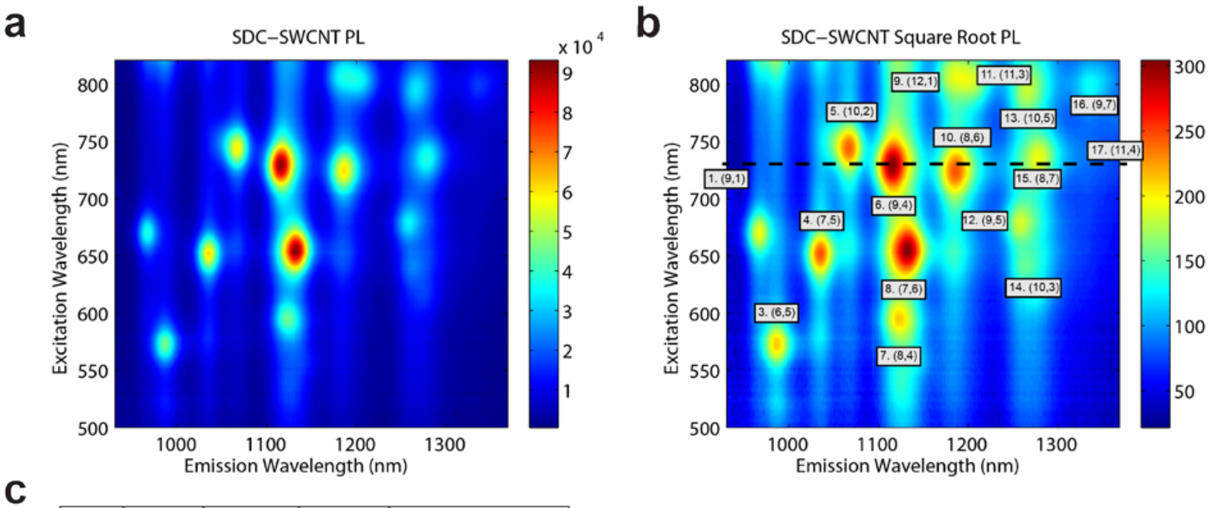

c

Group	(n,m)	Start (nm)	End (nm)	k-means seed (nm)
1	(9,1)	925	935	930
2	(8,3)	959	973	966
3	(6,5)	980	994	987
4	(7,5)	1029	1043	1036
5	(10,2)	1055	1072	1063.5
6	(9,4)	1105	1119	1112
7	(8,4)	1119	1127	1123
8	(7,6)	1127	1142	1134.5
9	(12,1)	1172	1179	1175.5
10	(8,6)	1179	1195	1187
11	(11,3)	1197	1210	1203.5
12	(9,5)	1251	1259	1255
13	(10,5)	1259	1266	1262.5
14	(10,3)	1266	1272	1269
15	(8,7)	1277	1294	1285.5
16	(9,7)	1330	1343	1336.5
17	(11,4)	1373	1385	1379

Figure S5. **Identification of nanotube chiralities in an SDC-dispersed HiPco sample**. **a**, A photoluminescence excitation/emission (PL) plot of SDC-HiPco nanotubes in solution. **b**, A PL plot of the square root of intensity was used to maximize contrast for assigning (n,m) chiralities. **c**, Tabulated peak emission wavelengths picked from the SDC-HiPco solution PL plot used as initial cluster bounds in a one-dimensional k-means clustering algorithm.

Sorting the nanotube emission peaks in ascending wavelength order allowed the separation of individual nanotube species, either by obvious gaps in the wavelength axis or via a one-dimensional k-means clustering algorithm[4]. The exercise resolved 17 (n,m) nanotube species (imaged on a glass surface in the absence of liquid) and provided an absolute count of the nanotube population. Separated populations were assigned (n,m) values based on an empirical Kataura plot[5].

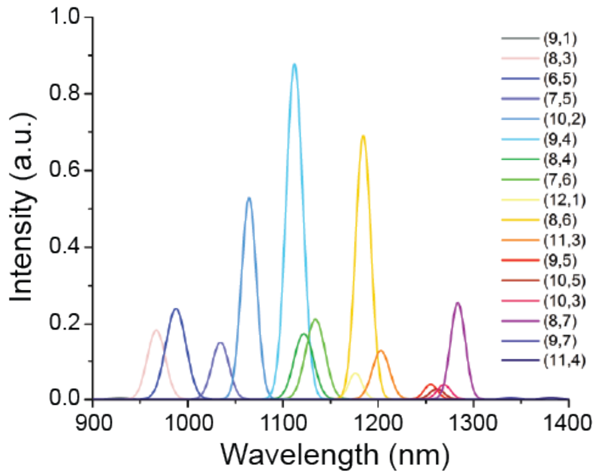

Figure S6. **Comparing the sum of individual nanotube spectra to the bulk solution spectrum**. A Gaussian curve was generated for each of 17 nanotube emission bands, calculated from the mean wavelength and FWHM acquired from multiple emission spectra of single nanotubes. Each Gaussian is scaled by the relative population count of the corresponding chirality.

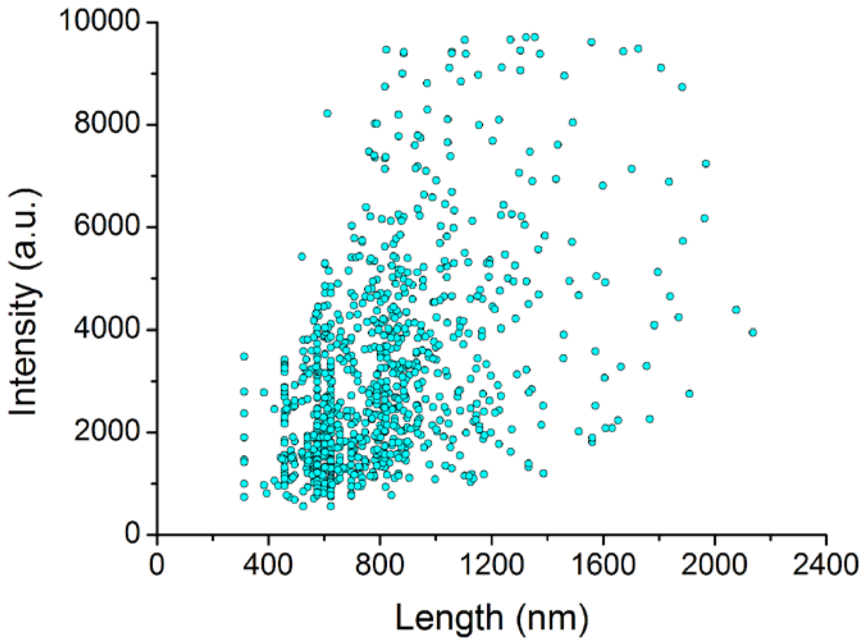

Figure S7. **Dependence of emission intensity on nanotube length.** The length (in nanometers) of 968 individual SDC-HiPco nanotubes plotted against the mean intensity from the nanotube showed a positive Spearman correlation of 0.4952, with $p < 0.001$.

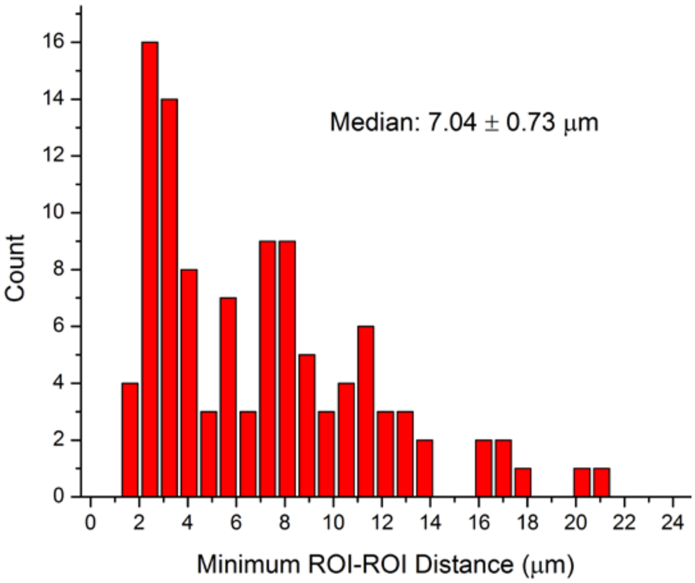

Figure S8. **A histogram of the nearest-neighbor distance for each fluorescent ROI detected in a hyperspectral cube of nanotubes endocytosed by live HeLa cells**.

SDC-encapsulated HiPco nanotubes incubated with HeLa cells, at a concentration of 1 mg/L, appeared as distinct fluorescent objects in a hyperspectral cube. The location of the centroid for each ROI was used to calculate the distance to its nearest ROI. A histogram of this nearest-neighbor distance indicates a median distance of 7 μm, significantly larger than the diffraction limited image size of 0.8 μm. This indicates that nanotube-containing ROI's were spatially well-separated and that the likelihood of two adjacent endosomes being counted as one ROI was minimal.

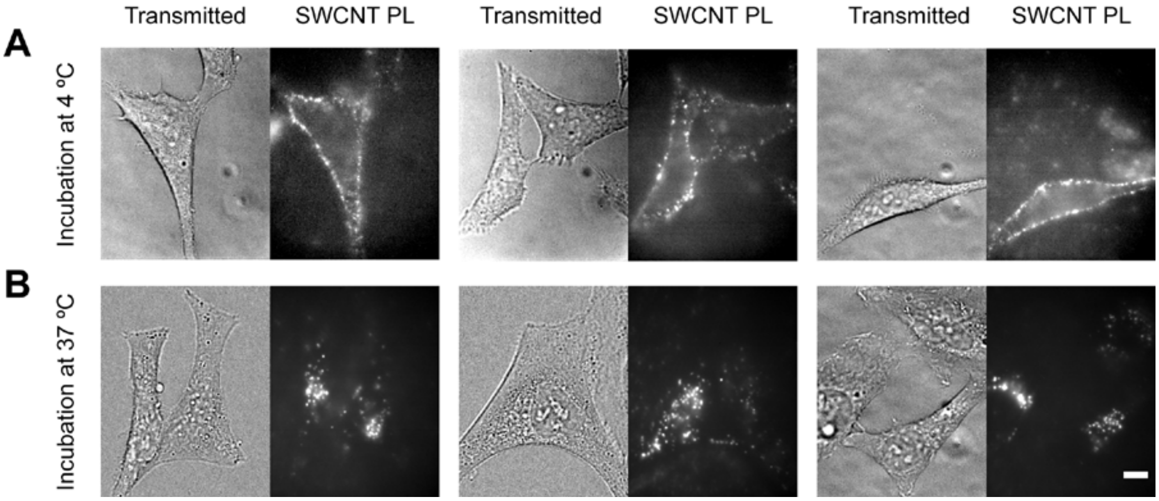

Figure S9. **Effect of temperature on nanotube uptake into HeLa cells**. **a**, SDC-encapsulated nanotubes incubated with HeLa cells at 4˚C. **b**, Incubation with nanotubes at 37˚C. Scale bar, 10 μm.

Nanotubes were incubated with HeLa cells for 30 minutes at 4˚C, rinsed of free nanotubes, and imaged using nIR fluorescence and transmitted light. The low-temperature incubation prevented the internalization of SDC-encapsulated nanotubes into HeLa cells, as confirmed by the localization of nanotubes on the outer plasma membrane on the cells at 4 hours. At 37˚C, the nanotubes were found throughout the interior of the cells. This confirms an energy-dependent mode of endocytosis, in agreement with previous studies[6].

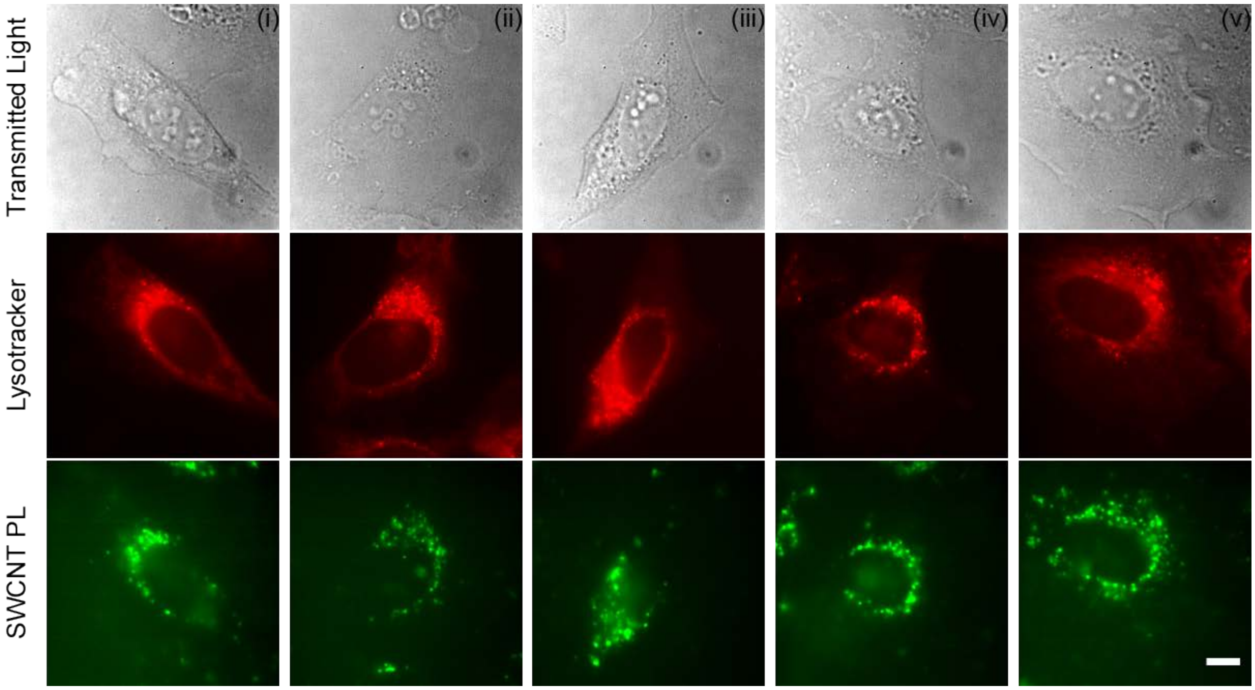

Figure S10. **Imaging SDC-dispersed nanotube emission and lysosomes**. Images of HeLa cells incubated with SDC-HiPco nanotubes and stained with LysoTracker dye. Transmitted light, nIR, and visible fluorescence images were acquired 6 hours after incubation with nanotubes. Scale bar, 10 µm.

Images of HeLa cells labeled with LysoTracker dye (Deep Red LysoTracker (ex/em 647/668 nm)) were obtained using a QIMAGING CCD camera attached to the hyperspectral microscope. Brightfield and nIR emission images in the same cells were obtained using the InGaAs camera on the same microscope. The two cameras had different aspect ratios and pixel sizes. Images were scaled to the same final image size and cropped to represent the same sample area. Emission from the SWCNT is localized within the same regions as the emission from Lysotracker in all five representative images shown.

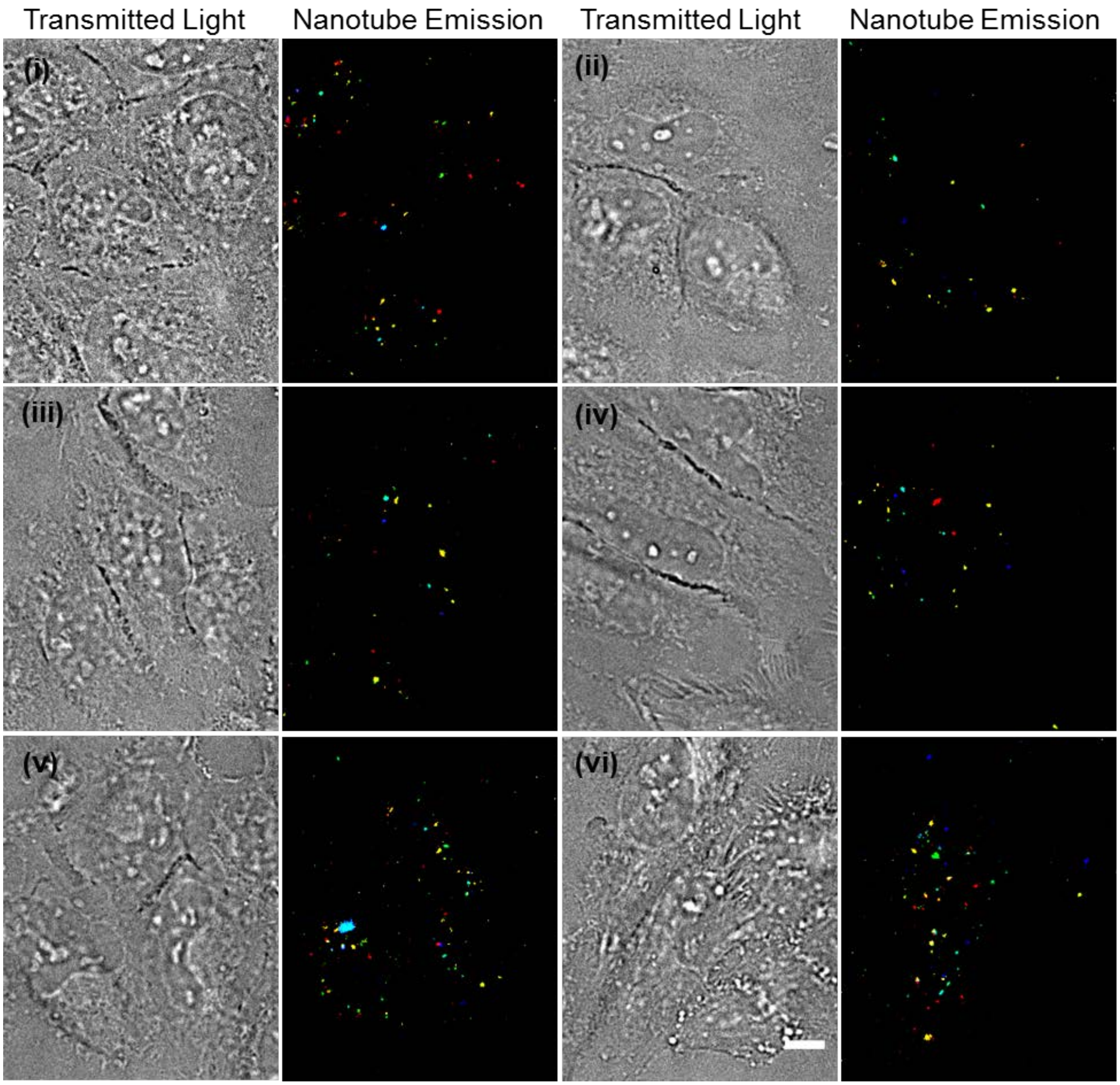

Figure S11. **False-colored images of multiple nanotubes (n,m) species in live HeLa cells**. SDC-HiPco nanotubes, at 1 mg/L, were exposed to HeLa cells in media with serum for 30 minutes. Transmitted light and spectrally resolved nIR nanotube emission images are shown with colors corresponding to the coloring scheme in Figure 2 of the main text. Scale bar, 10 µm. The z-planes were chosen by maximizing the number of visible ROIs.

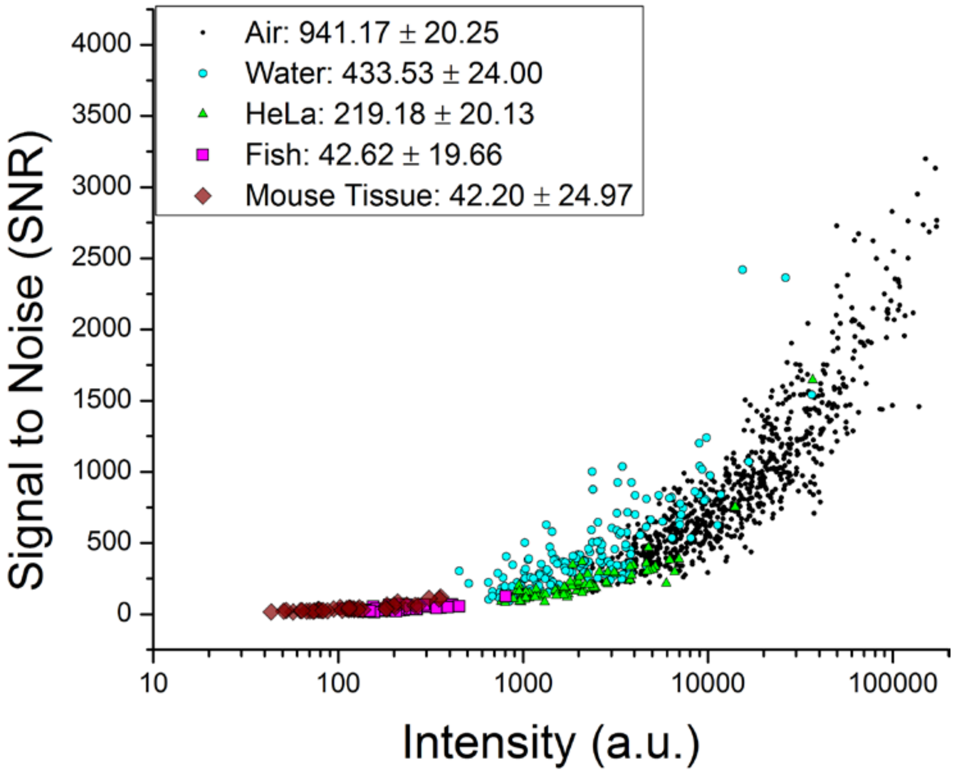

Figure S12. **Calculated signal-to-noise ratio of nanotubes, compared across the examined media.** The relative SNR of nanotubes in: Air (surface) > Water (surface) > HeLa > Fish ≈ Mouse.

Table S1. Time required for hyperspectral cube acquisition. Hyperspectral cubes were acquired from 900-1400 nm.

Exposure Time (s)	Frames	Unrectified Frames	Acquisition Time (min:sec)
0.05	126	152	00:20
0.1	126	152	00:32
0.5	126	152	2:03
1	126	152	3:52
2	126	152	6:32
4	126	152	10:30

Table S2. Measurement of excitation laser polarization. A linear polarizer was placed in the excitation path of the hyperspectral microscope and rotated in increments of 30 degrees. The total power at the sample was measured without an objective in the optical path.

Angle of Linear Polarizer	Power (mW)
0	520
30	510
60	510
90	510
120	525
150	522
180	520

Table S3. Chirality assignment of SDC-dispersed nanotubes in HeLa cells. Tabulated peak emission wavelengths picked from the SDC-HiPco solution PL plot to be used as initial cluster bounds in a one-dimensional k-means clustering algorithm.

Group	(n,m)	Start (nm)	End (nm)	k-means seed (nm)
1	(8,3)	960	975	967.5
2	(6,5)	984	999	991.5
3	(7,5)	1027	1055	1041
4	(10,2)	1057	1078	1067.5
5	(9,4)	1103	1116	1109.5
6	(8,4)	1116	1128	1122
7	(7,6)	1128	1146	1137
8	(12,1)	1175	1187	1181
9	(8,6)	1188	1197	1192.5
10	(11,3)	1200	1215	1207.5
11	(9,5)	1270	1282	1276
12	(10,5)	1282	1296	1289
13	(8,7)	1298	1308	1303

References:

1 Schaffer, B. S. *et al.* Immune competency of a hairless mouse strain for improved preclinical studies in genetically engineered mice. *Molecular cancer therapeutics* **9**, 2354-2364 (2010).

2 White, R. M. *et al.* Transparent Adult Zebrafish as a Tool for In Vivo Transplantation Analysis. *Cell Stem Cell* **2**, 183-189 (2008).

3 Nusslein-Volhard, C. & Dahm, R. *Zebrafish*. (Oxford University Press, 2002).

4 Seber, G. A. F. *Multivariate Observations*. (Wiley, 2004).

5 Ghosh, S., Bachilo, S. M. & Weisman, R. B. Advanced sorting of single-walled carbon nanotubes by nonlinear density-gradient ultracentrifugation. *Nature Nanotech* **5**, 443-450 (2010).

6 Bhattacharya, S., Roxbury, D., Gong, X., Mukhopadhyay, D. & Jagota, A. DNA Conjugated SWCNTs Enter Endothelial Cells via Rac1 Mediated Macropinocytosis. *Nano Lett.* **12**, 1826-1830 (2012).
